# Factors affecting residents’ internal motivation, grit, and well-being

**DOI:** 10.1186/s12909-023-04679-2

**Published:** 2023-10-19

**Authors:** Pimwipa Chuented, Pongtong Puranitee, Samart Pakakasama, Suganda Meepanya

**Affiliations:** Department of Pediatrics, Faculty of Medicine, Ramathibodi Hospital, Mahidol University, Bangkok, Thailand

**Keywords:** Grit, Internal motivation, Medical education, Residents, Well-being

## Abstract

**Background:**

Residents completing competency-based medical education for postgraduate training face many challenging situations that may compromise their well-being or result in exhaustion or burnout. Factors described in self-determination theory and grit are important for residents’ achievement of learning outcomes and well-being. This study explored the relationships among internal motivation, grit, well-being, and related factors among non-Western Asian residents.

**Methods:**

We conducted an explanatory sequential mixed-methods survey-based study to explore correlations among satisfaction with basic psychological needs, grit, and well-being from September to November 2021 among residents at Ramathibodi Hospital, Mahidol University, Thailand. Data were collected with the Basic Psychological Needs Scale, Short Grit Scale, and World Health Organization-Five Well-Being Index. Next, participants with the highest and lowest scores for each scale were purposively invited to participate in semi-structured interviews. Interview data underwent thematic analysis and data collection continued until saturation was reached.

**Results:**

In total, 245 residents (51% major ward, 65% female) completed the survey. There were strong associations between internal motivation, grit, and well-being (r = 0.46–0.90). Female residents had higher autonomy and relatedness scores than males (p = 0.04 and p = 0.01, respectively), and residents with less family responsibility had higher relatedness scores than other residents (p = 0.01). Residents who got more sleep had higher autonomy, relatedness, and well-being scores than those that slept less (p < 0.05). Residents who exercised > 5 times/week had higher well-being scores than other residents (p < 0.01). Thirty residents completed interviews. The thematic analysis revealed internal motivation, grit, and well-being were promoted by a supportive learning environment, a well-designed curriculum, actions and personalities of faculty members, and good personal factors.

**Conclusion:**

Internal motivation is significantly correlated with residents’ grit, well-being, gender, family burdens, exercise, and sleep hours. Priority should be given to promoting internal motivation, grit, and well-being among residents by enhancing a positive learning environment, creating well-designed curricula, fostering good characteristics and actions among faculty members, and supporting residents’ personal lives.

**Supplementary Information:**

The online version contains supplementary material available at 10.1186/s12909-023-04679-2.

## Introduction

Residents completing education during workplace training often face challenging situations, which may compromise their well-being and lead to exhaustion or burnout. Challenges that can lead to burnout include time limits, intense patient care pressure, feeling insecure in one’s knowledge or abilities when performing non-physician activities, and life crises [1–4]. Motivation is critical to create self-determined behavior to achieve learning goals and overcome difficult situations. Based on a sophisticated psychological theory of human conduct, self-determined behavior is defined as independent behavior via self-acceptance, self-esteem, and self-love [5]. This suggests that people have the capacity to evolve, expand, and incorporate development processes into their own identities. Deci and Ryan hypothesized that self-determination is developed through the satisfaction of three fundamental needs—autonomy, relatedness, and competence [5]. The need for autonomy is defined as the desire for the freedom to make decisions for oneself. The need for relatedness is defined as the desire to have relationships with others. Finally, the need for competence is defined as the desire to feel effective in actions. Internalization refers to the internal motivation developmental process that is required to satisfy these three fundamental needs [6]. Furthermore, fostering motivation for learning depends on the learning environment. A key factor related to the external environment is encouragement from friends, loved ones, and family [7]. However, such encouragement may also be a drawback.

In the presence of adverse external circumstances, internally motivated residents are more likely to learn and perform better than other residents, and they may also experience a greater level of life satisfaction. In the medical school context, stress is a barrier to learning that can be caused by various factors, including time constraints, academic pressure, and a lack of adequate sleep. However, few studies have investigated variables that affect internal motivation among residents in both Western and non-Western contexts. Some research has noted that women have a greater propensity to achieve relatedness (a component of internal motivation) than men [8], whereas other studies claimed that gender, age, or medical school year had no significant effects on need fulfillment characteristics [9].

Grit is a key component of both academic success and overall well-being [10]. The term “grit” describes an individual’s ongoing dedication and effort toward achieving their long-term goals. It is defined by the ideas of enthusiasm and tenacity, without which a person’s life cannot be successful [11]. The three components of self-determination theory along with grit are key characteristics residents must pursue to attain positive learning outcomes and well-being. However, there are some value, context, religion, and cultural distinctions related to these concepts between Western and non-Western countries. In Eastern collectivistic cultures, social harmony is prioritized over the individual’s goals and values, whereas Western cultures emphasize the importance of the individual’s goals and values [12]. A previous study noted that grit supports students’ capacity to adapt to their changing academic environments [9]. In particular, grit denotes the ability to respond flexibly to new challenges originating from unforeseen circumstances, and includes evidence of persistence, willingness to experience/accept failure, determination, and hard work [13]. However, grit is not simply “resilience in the face of failure”; rather, it is a mechanism that facilitates the formation and maintenance of the profound commitment required to achieve individual goals [8].

In Eastern conceptualization, well-being is understood as the result of the actions of a socially embedded person whose identity implies the existence of a social context, rather than as a result of the actions of an isolated individual. Therefore, social action aimed to enhance the well-being of individuals must acknowledge the significance of the social context. The promotion of well-being should not be individualistic, but rather social [14, 15].

The present study sought to clarify the critical elements influencing residents’ motivation in relation to the three basic needs described in self-determination theory (autonomy, relatedness, and competence) and grit. However, studies focused on residents’ self-determination behavior in non-Western Asian contexts are limited. Therefore, the following research questions guided this study.

- What factors (e.g., grit, well-being) are related to residents’ self-determination behavior?
- What do residents perceive as the key factors influencing internal motivation regarding self-determination theory and grit?
- What are the relationships between internal motivation, grit, well-being, and other factors among non-western Asian residents?

## Methods

This study used an explanatory sequential cross-sectional mixed methods design comprising subsequent quantitative and qualitative phases. Data were collected with questionnaires and semi-structured individual interviews in the quantitative and qualitative phases, respectively. This study was conducted between September and November 2021 among residents in all departments of Ramathibodi Hospital, Mahidol University, Bangkok, Thailand. This study was approved by the Ethics Committee of the Faculty of Medicine of Ramathibodi Hospital, Mahidol University (ID 1416).

### First (quantitative) phase

In the quantitative phase, data were collected with an electronic survey, which aimed to identify participating residents’ satisfaction in terms of their basic psychological needs, grit, and well-being. The survey was disseminated to all residents from first to senior year (N = 700). Participants provided informed consent for confidential data collection, which only the researchers could access, and were informed that they were free to withdraw from the study if they wished. The correlation coefficient (r) used to calculate the required sample size of 8.27 to 133.86 participants based on existing research [16] ranged from 0.24 to 0.84.

### Instruments

The framework for this study comprised basic psychological needs, grit, and well-being; therefore, data were collected with the Basic Psychological Needs Satisfaction Scale, the Short Grit Scale (Grit-S), and the World Health Organization-Five (WHO-5) Well-Being Index. These three questionnaires were administered in the first phase of this study.

### Basic Psychological needs satisfaction scale

The original form of this scale was developed by Deci and Ryan. The scale has 21 items on three subscales (autonomy, competence, and relatedness), with responses on a 7-point Likert scale [5, 17, 18]. Autonomy refers to when an individual feels like the initiator of their own actions and experiences. Competence is the perception of optimally accomplishing challenging tasks and achieving the desired result. Relatedness refers to a sense of caring, reliance, and mutual respect for others. These factors are considered to be essential for personal growth and internal motivation [5]. The Cronbach’s alpha coefficients for the autonomy, competence, and relatedness subscales were 0.71, 0.60, and 0.74, respectively. The overall Cronbach’s alpha was 0.83. We used the Thai version of the scale [19], which showed good reliability with Cronbach’s alpha values of 0.86 for the whole scale, 0.78 for the autonomy subscale, 0.65 for the competence subscale, and 0.78 for the relatedness subscale.

### Grit-S

This two-factor structured questionnaire was adapted from the original Grit Scale [11] with four fewer items and improved psychometric properties. A previous study demonstrated evidence that the Grit-S had good internal consistency, retest stability, consensual validity with informant report versions, and predictive validity [20]. The scale has eight items with responses on a 5-point Likert scale. The Cronbach’s alpha values were 0.63 for the consistency of interest factor and 0.60 for the perseverance of effort factor [21]. The Grit-S was translated and adapted to fit the Thai medical school context, with a Cronbach’s alpha value for the total scale of 0.96 [22].

### WHO-5 well-being Index, Thai version (WHO-5-T) [23]

The WHO-5 Well-being Index was translated into Thai for a cross-cultural adaptation, including forward-translation, synthesis of the translation, backward-translation, cross-cultural adaptation, and pilot testing. The WHO-5-T showed good internal consistency (Cronbach’s alpha coefficient = 0.87) and sufficient discriminatory validity as a self-report screening tool for detecting major depression. At a standard cut-off point of < 12, the WHO-5-T had the best sensitivity (89%) and specificity (71%) [23]. The WHO-5-T is a short and quick self-report tool for detecting depression in the Thai language. It comprises the same five items as the original English version. Each item of the WHO-5-T is score of 5 to 0.

### Second (qualitative) phase

Based on the results of the first phase, 30 residents with the highest and lowest scores for internal motivation, sense of passion, and perseverance in residents’ life were purposively invited to participate in the second phase. Miles and Huberman’s theories [24] suggest that choosing individuals with high or low scores for each set of questionnaires is necessary to gain sufficient insights and establish a clear key point from the research. Depending on information saturation, 20–30 participants may be invited to complete an individual interview. In the qualitative part of this study (second phase), semi-structured individual interviews were conducted to refine and explore factors promoting motivation, grit, and well-being among residents in more depth than in the first phase [25].

As it was considered a sensitive topic, an individual interview approach was used to collect data. The interviews were conducted by a researcher who had experience with qualitative interviews. A list of questions was developed to explore residents’ experiences influencing (positive and negative) their internal motivation, their sense of passion and perseverance, and their well-being in medical school. The interviews were audio recorded without labeling or collecting any identifiable information from participants. The audiotape file was deleted immediately after transcription. The interviewer was a pediatric resident who had no authority over the participants.

### Data analysis

In the quantitative phase, descriptive analyses were conducted, including mean comparisons based on different factors and correlation analysis. Pearson’s correlation coefficients were used to identify the correlations among sense of internal motivation and sense of passion and perseverance and well-being among participating residents using SPSS version 18.0 (Mahidol license, Bangkok, Thailand). Univariate and multivariate regression analyses were conducted, and the coefficients and 95% confidence intervals were reported.

The second phase involved thematic analysis [26] in which the generally accepted principles of open coding, axial coding, and selective coding were applied to the data [27] To explore topics and aspects newly mentioned in later interviews, this analysis was conducted iteratively while data collection was still in progress. The transcripts from the interviews were analyzed independently by two researchers until theoretical data saturation was reached. In cases of disagreements in interpretation, these researchers discussed the results until consensus was reached. A presentation of descriptions and themes was carefully translated into English. Statistical significance was set at p < 0.05.

## Results

### First (quantitative) phase

#### Participants’ demographic characteristics

Of the 700 invited residents, 245 (response rate 35%; 65% female) residents participated in this study. Participants’ demographic characteristics are shown in Table 1.

**Table 1:** Participants’ baseline characteristics (N = 245)

characteristic	n	%
gender		
male : female	84 : 161	35 : 65
**age, years**		
25–30 30–35 > 35	228 15 2	93 6.1 0.9
**status**		
single : married	234 : 11	95.5 : 4.5
**family burden as resident (perception)**		
low level moderate level high level	162 69 11	67 28.5 4.5
**alcohol**		
drinker	51	21
**department**		
major : minor	125 : 120	51 : 49
**year of resident**		
1st	73	30
2nd	105	43
3rd	60	25
$\ge$4th	7	2
**sleep time**		
< 35 h/week	43	17.6
$\ge$35 h/week	202	82.4
**exercise time**		
never	96	39
> 5 h/week	9	4
1–5 h/week	140	57

The univariate and multivariate analyses showed there were significant relationships between autonomy and gender, sleep time, and exercise time. Gender and family burden were significantly associated with competence. Sleep time and exercise were significantly associated with residents’ well-being (Supplement file).

#### Factors related to basic psychological needs, grit, and well-being among residents

Gender was significantly associated with autonomy (p = 0.04) and relatedness (p = 0.01) (Table 2.1), with female residents showing more autonomy and a greater sense of relatedness than male residents. Family burden was significantly associated with relatedness (p = 0.01) (Table 2.2) as residents with less family responsibility scored higher on relatedness than other residents. Exercise time was significantly associated with autonomy (p = 0.04), relatedness (p = 0.02), and well-being (p < 0.001) (Table 2.3). Residents who never exercised had lower scores for autonomy and relatedness than those who exercised 1–5 times/week. Those who exercised > 5 times/week had higher scores for well-being than other residents. Sleep was significantly associated with autonomy (p = 0.01), relatedness (p = 0.04), and well-being (p < 0.001) (Table 2.4). Residents who had a sleep time of > 35 h/week had higher scores for autonomy, relatedness, and well-being than other residents.

**Table 2.1:** Gender factors (mean ± standard deviation)

	male (n = 84)	female (n = 161)	p-value
**autonomy**	3.16 ± 0.99	3.43 ± 0.98	0.04*
**competence**	3.17 ± 1.23	3.33 ± 1.17	0.31
**relatedness**	3.9 ± 1.04	4.23 ± 0.98	0.01*
**grit**	2.23 ± 0.81	2.33 ± 0.76	0.34
**well-being**	14.77 ± 4.02	15.52 ± 3.78	0.15

*p < 0.05

**Table 2.2:** Family burden factors (mean ± standard deviation)

	low level (n = 162)	moderate level (n = 69)	high level (n = 11)	p-value
**autonomy**	3.41 ± 1.03	3.23 ± 0.95	2.84 ± 0.51	0.1
**competence**	3.38 ± 1.24	3.14 ± 1.12	2.67 ± 0.74	0.08
**relatedness**	4.23 ± 1.05	3.94 ± 0.92	3.51 ± 0.63	0.01*
**grit**	2.33 ± 0.81	2.22 ± 0.72	2.25 ± 0.8	0.6
**well-being**	15.49 ± 3.91	14.68 ± 3.78	14.55 ± 3.69	0.29

*p < 0.05

**Table 2.3:** Exercise time factors (mean ± standard deviation)

	never (n = 96)	> 5 h/week (n = 9)	1–5 h/week (n = 140)	p-value
**autonomy**	3.14 ± 0.9	3.59 ± 0.77	3.46 ± 1.05	0.04*
**competence**	3.06 ± 1.08	3.33 ± 0.96	3.43 ± 1.26	0.06
**relatedness**	3.9 ± 0.99	4.34 ± 0.96	4.25 ± 1.01	**0.02**
**grit**	2.18 ± 0.71	2.51 ± 0.72	2.36 ± 0.82	0.16
**well-being**	14.42 ± 3.72	20.11 ± 2.71	15.54 ± 3.79	< 0.001*

*p < 0.05

**Table 2.4:** Sleep time factors (mean ± standard deviation)

	< 35 h/week (n = 43)	≥ 35 h/week (n = 202)	p-value
**autonomy**	3 ± 1.03	3.41 ± 0.97	0.01*
**competence**	3.04 ± 1.24	3.33 ± 1.18	0.14
**relatedness**	3.84 ± 0.91	4.18 ± 1.02	0.04*
**grit**	2.21 ± 0.84	2.32 ± 0.77	0.4
**well-being**	13.56 ± 4.56	15.63 ± 3.62	< 0.001*

*p < 0.05

#### Correlations between internal motivation, grit, and well-being

Internal motivation (which included autonomy, competence, and relatedness) was strongly positively associated with grit (autonomy: r = 0.846; competence: r = 0.906; relatedness: r = 0.842; p < 0.001). Residents who demonstrated a strong passion and perseverance were more internally motivated to achieve their goals. In addition, autonomy, competence, and relatedness were positively correlated with well-being (r = 0.625, r = 0.510, and r = 0.602, respectively; p < 0.001). Grit was mildly positively correlated with residency well-being (r = 0.464; p < 0.001) (Table 3).

**Table 3:** Correlations between internal motivation, grit, and well-being in residency

		internal motivation			grit
		autonomy	competence	relatedness	
**grit**	**pearson’s correlation**	0.846	0.906	0.842	1
	**p-value**	< 0.001*	< 0.001*	< 0.001*	
**well-being**	**pearson’s correlation**	0.625	0.510	0.602	0.464
	**p-value**	< 0.001*	< 0.001*	< 0.001*	< 0.001*

*p < 0.05

### Second (qualitative) phase

In total, 30 residents were interviewed; 18 from major departments, including pediatrics, surgery, internal medicine, orthopedics, and obstetrics and gynecology, and 12 from minor departments, including emergency medicine, anesthesia, otolaryngology, family medicine, pathology, radiology, and psychiatry. The interviewed residents had high and low scores for basic psychological needs, grit, and well-being. The main themes encompassing factors promoting internal motivation, grit, and well-being that emerged from the thematic analysis were classified in four domains: supporting learning environment, faculty member characters and action in teaching, curriculum design, and personal factors outside the workplace.

#### Creating a positive learning environment

Residents described a positive learning environment as one in which they: felt appreciated and respected; had strong relationships; and received support from faculty members, fellow residents, peers, seniors, juniors, other professionals, and patients. Teachers played a crucial role in fostering greater autonomy by providing residents with care and assistance and listening to residents in an approachable and less hierarchical environment. In this way, they encouraged residents to be authentic, feel more at ease and self-assured when expressing their opinions, and make decisions regarding patient care without criticizing others.

*“Compared to institutions that had previously graduated, Ramathibodi Hospital’s teachers were very open-minded to residents’ voices and thoughts. There was no consult protocol based on seniority of the residents and teachers. I felt more confident and have freedom to voice my own opinion. No concern or overstress about being blamed when there were some mistakes.”* (ID24, female resident, year 3).

*“I appreciated the teachers for allowing me to discuss about as I wanted…Being a resident here, I truly like it. Instead of criticizing my mistakes, the teacher suggests an alternate decision making, and better options for patient care.”* (ID07, female resident, year 1).

“*A great work atmosphere resulted from everyone respecting one another and refraining from using derogatory words or displaying anger against one another.”* (ID30, male resident, year 2).

Patients’ negative reactions toward residents without adequate protection from the supervision system could compromise residents’ perception of a safe learning environment and had a detrimental impact on their autonomy.

*“During [the] COVID-19 situation, when I was assigned to postpone patients’ appointments to elective surgery, that had been canceled multiple time and postponed indefinitely. [A] Patient who had their appointment canceled was suddenly angry with me and used harsh words to me, then he threatened to sue me and request the sound recording accompanying the lawsuit…made me feel bad that no one can protect me. Although, I understood the feeling of [the] patient, but I felt insecure in work.”* (ID26, male resident, year 1).

Furthermore, an act of disrespect could compromise the good relationships between senior doctors and resident and have negative impact on teamwork.

*“I felt bad when a fellow doctor (sub-specialty trainee) used strong reprimands, including throwing used gloves. Even though I realized I made a mistake by delaying consulting him in an emergency situation, I also believed the senior action was inappropriate. This makes me reluctant to interact with this fellow doctor again.”* (ID22, male resident, year 2).

Residents valued challenging tasks to achieve high expectations, although adequate support from teachers or friends was necessary to meet these expectations.

*“I liked when people pose challenging queries. Even though I couldn’t answer the question, I enjoyed being with my friends, helping each other think and solving difficulties together.”* (ID08, female resident, year 1).

Positive feedback and well-deserved rewards encouraged residents to strive for greater self-improvement, which had a positive effect on their grit.

*“I had received compliments on my earlier research presentations. It inspired me to finish this research.”* (ID25, female resident, year 3).

*“When I presented case report, I assumed that no one would be interested in listening too much. But the teacher still listened attentively and commented positively. Keep giving advice and encouragement.”* (ID05, female resident, year 1).

*“I felt good doing challenging work. Even though I would feel discouraged while working, everyone around me, especially the teacher, offered suggestions and support until the project was successful.”* (ID17, resident, year 2).

#### Faculty member characters and action in teaching

Faculty members’ ability to provide constructive feedback, act as role models, demonstrate mutual respect, be approachable, and be open-minded were cited as important factors that influenced residents’ internal motivation, grit, and well-being. In addition, longitudinal support from faculty members along with good relationships could enhance residents’ grit in relation to achieving their long-term goals.

*“I was grateful for the time I had set up for student feedback without restriction. Having teachers’ pay attention was a positive thing. Can assist in problem resolution.”* (ID10, female resident, year 2).

“*When people praised me, I felt accomplished. The award was more proof of my success. Encouraged me in maximizing my possibilities for profession. Teachers who are accessible could be able to talk through difficult issues until they are resolved. Additionally, friends and elders provide helpful examples of how to handle issues increasing work experience.”* (ID06, female resident, year 3).

*“I appreciated always having a teacher consultant. The teacher made suggestions and assisted with problem-solving, this could help to keep up my task until it was completed.”* (ID27, female resident, year 1).

#### Curriculum design

Residents were willing to learn if they had clear learning goals and objectives with meaningful or useful tasks, clear role assignments to reduce conflict while working together, tasks that were sufficiently challenging for their skill level, a learning timetable that fit with the flow of healthcare services and work, and chances to socialize and get to know each other.

In terms of autonomy, residents indicated that a well-designed curriculum should consider students’ ability to catch up and adapt to new information so they could apply it in practical situations, and not merely focus on teaching practice and the quality of their lessons. As a result, learning objectives should be agreed upon with the teacher and connect to the individual resident’s level.

*“During the residency training program that [a] senior resident [should] supervise [the] novice first year resident, making decisions only [on] uncomplicated issues, then a second-year resident will have an opportunity to approach and manage more complex cases under [their] teacher’s supervision. This systematic role arrangement which match with the level of difficulty or complexity made me feel more confident to make decision.”* (ID17, female resident, year 2).

In terms of relatedness, residents indicated student relationships suffered because of the competitive learning and assessment system.

*“A poor educational system, in my opinion, that prioritized grades and performance. Teachers would pay special attention to residents who scored highly or had exceptional abilities. Ignoring the importance of acting morally uprightly but this isn’t like that. It’s great not to be in a hostile environment.”* (ID16, male resident, year 1).

A resident’s sense of lacking knowledge occurred as a result of the poor allocation of time between practice and learning activities, which limited their capacity for autonomous decision-making.

*“I had no time for class when I was on the emergency rotation since there were so many patients. Due to my inexperience of the proper order and the fact that my directions appeared odd to my peers, the nurse refused to follow my instructions. All of which made me reduced autonomy to make a decision from insecure perception of knowledge.”* (ID08, female resident, year 1).

#### Personal factors outside the workplace

Residents’ grit, well-being, and internal motivation were influenced by their perceptions of their ability to manage their lives effectively, such as adequate personal time, their financial situation, and the support and understanding of their friends and family. For example, residents reported that adequate relaxation time was required to recharge one’s spirits and improve their well-being.

*“I really believed that having free time so you may take charge of your own life was essential for being able to work happily. How much fun will the course be? It’s undoubtedly not enjoyable if you did have no time for yourself.”* (ID19, male resident, year 3).

*“In 3 weeks, I had to work 10 shifts, I felt depressed every time when I was on duty.”* (ID08, female resident, year 1).

Each resident had different goals they wanted to achieve in their studies and life, such as making money, seeing patients’ recovery, and receiving a graduation certificate. Residents’ clear goals and determination to achieve their goals were reported to be crucial factors affecting their grit. In addition, strong support from family and friends was reported as a factor that could help residents to maintain their grit and pursue their goals.

*“One of the hardest things in my life was completing a research study. I had given up on three research projects, which left me dejected. I don’t want to do the research anymore, but I was unable to stop. Because I wouldn’t graduate if I couldn’t complete it. To get over this, I tried to soothe myself and ask my professors, friends, and family for guidance.”* (ID02, female resident, year 3).

## Discussion

In accordance with the explanatory sequential mixed-method approach used in this study, the findings related to the research questions are discussed sequentially below to elucidate the key factors that influenced internal motivation and grit and related to well-being among residents.

This study revealed that internal motivation, grit, and well-being were positively correlated, with grit showing the strongest association with internal motivation. This was consistent with self-determination theory. These findings were also similar to previous studies from Turkey [16] and Canada [28], which concluded that positive well-being was directly associated with internal motivation and grit. This may reflect the collectivist Eastern culture, where well-being is conceptualized as a social influence [14, 15], and could explain the relationship between residents’ relatedness in motivation and their perception of well-being. These findings were also consistent with those of a previous study conducted among orthopedic physicians in the United States [29, 30] that found a negative relationship between grit and burnout and positive relationships between grit and personal accomplishment and well-being. That study also claimed that surgeons with 21 or more years of experience had higher grit and lower burnout scores, and married physicians reported high satisfaction. However, other studies reported different results from our study. For example, a Thai study focused on grit, motivation, and anxiety found anxiety was positively correlated with internal motivation and negatively correlated with grit, although this was not statistically significant [29]. The present study revealed that female gender, family issues, exercise time, and sleep time influenced residents’ internal motivation, grit, and well-being. Females were reported to have more social skills than males, which was reflected in higher scores on the relatedness subscale for meeting basic needs. This assumption was consistent with previous research [16]. Our finding related to sleep time was consistent with a Malaysian study involving undergraduate students in which sleep quality was significantly related to grit [31]. Previous research found no evidence for the family burden and exercise time factors. Furthermore, only family members influenced the motivation of adult patients with chronic diseases [32]. However, having sufficient time to deal with personal matters influenced their quality of life and indirectly promoted internal motivation.

The secondary objective of this sequential mixed methods study was to identify the critical factors that influenced internal motivation, grit, and overall well-being from the perspective of residents. These findings suggested that the learning environment was perceived as the crucial factor influencing residents’ internal motivation, well-being, and grit. The learning environment was previously reported to be an important context, with psychosocial and material dimensions [33]. A resident’s environment includes their classrooms, seniors, and teachers, as well as coworkers in other professions (e.g., nurses, clerks, spouses/housemates, and patients and patients’ families). Therefore, different people in residents’ learning environment were considered in relation to internal motivation, grit, and well-being. Some studies also referred to the physician’s family and close contacts as part of their learning environment [34]. Residents’ learning occurs through participation in work, which can be understood in terms of how the workplace encourages or discourages individuals’ participation in work activities and access to direct and indirect guidance. Given the connection between participation and learning, a vital issue for understanding and improving workplace learning is how to invite individuals’ or groups’ participation [35]. Related theories, such as ecological psychology and workplace learning, emphasized that affordances in the environment with which learners actively engaged facilitated social interactions related to learning [35]. Our study found that the perception that they were in a good learning environment could help residents’ mental health and stimulate their learning. This was in contrast to a toxic learning environment in which there was a lack of effective communication or criticism of work (e.g., focusing on the main action and not listening to opinions), and residents felt unsafe and unstable while working and learning. This may impact their learning motivation and eventually result in feelings of being burned out in learning. This was consistent with a previous Thai study that found the learning environment was associated with burnout [4]. Furthermore, teacher-student relationships were important for learner motivation. High levels of teacher collaboration influenced students’ perceptions of assessment for learning. Interpersonal theory can be used to better understand assessment relationships [36]. Good teachers are characterized by acknowledgement, self-assurance, respect for others, empathy, justice, admiration, flexibility, rationality, interest, emotional intelligence, trustworthiness, humor, the ability to educate, and the capacity for participation, all of which allow them to positively impact their students [37, 38]. Residents in this study appreciated teachers who understood and allowed them to express their opinions. Furthermore, they reported they felt like “a part of the family” when teachers’ paid attention to details of their lives, which allowed for good relationship building. This is likely to impact students’ learning motivation. However, most residents disliked teachers that showed inattention, authoritarianism, hierarchy, and inflexibility.

It is also critical to inspire students to learn by providing them with an engaging curriculum. Curriculum design that provides motivation for learning and sets long-term goals for graduation should begin in childhood with enjoyable [39] or interesting [5] content. However, at higher education levels, students tend to be more concerned with performance assessment scores [40, 41]. This is a major source of motivation to enable students to achieve their goals. However, a previous study [42] found that many residents experienced burnout in learning and work, which caused a lack of motivation for learning and eventually failure in education. As well as a student’s eagerness to learn, many surrounding factors support intrinsic motivation to continue learning and achieve goals. This study emphasized the importance of having a student-centered curriculum design, clear roles and tasks that are appropriate for residents’ level of competency, a mentoring system, adequate personal time, and activities to foster relationships among students.

In summary, faculty development programs should focus on creating a positive learning environment and paying attention to the traits and actions of faculty members during their teaching. A curriculum design committee should focus on developing a quality curriculum and considering supporting residents’ personal lives.

### Strengths and limitations

Despite the limitation of the low response rate, the numbers of participants in the quantitative and qualitative phases of this study were adequate. The data drawn from the quantitative phase may be considered to represent all residents in Ramathibodi Hospital as our sample included residents in all levels and covered most departments. The mixed methods design of this study allowed us to qualitatively explore the trends and correlations revealed in the quantitative phase in more depth. This study could be replicated elsewhere to explore other contexts. However, as this study was conducted among residents from Ramathibodi Hospital, the generalizability to other contexts, such as other institutions or countries, was limited.

## Conclusions

Internal motivation has significant correlations with grit and well-being, which are influenced by gender, family issues, exercise time, and sleep time. The variables described above should be considered for the success of life and work among residents. Optimizing these elements should be a priority in curriculum design and the learning environment should be improved to match individual learning needs. In addition, having a clear purpose and allocating appropriate tasks to residents in each class year are critical elements that affect internal motivation, grit, and well-being.

### Electronic supplementary material

Below is the link to the electronic supplementary material.

Supplementary Material 1

## Data Availability

The datasets generated and analyzed during the present study are not publicly available due to confidentiality but are available from the corresponding author on reasonable request.
