# Supplementary material for: Factors affecting residents’ internal motivation, grit, and well-being
Source: BMC Med Educ. 2023 Oct 19;23:779. doi: 10.1186/s12909-023-04679-2 (PMC10588185; doi:10.1186/s12909-023-04679-2)
Supplement: Supplementary file 1 — Supplementary Material 1 [file 12909_2023_4679_MOESM1_ESM.docx]

Table 1. A univariate and multivariate analysis of the demographic factors and autonomy

| AUTONOMY | Univariate | | Multivariate | |
| --- | --- | --- | --- | --- |
|  | Coef. (95% CI) | P-value | Coef. (95% CI) | P-value |
| GENDER |  |  |  |  |
| FEMALE | 1.88 (0.04, 3.71) | 0.045 | 2.06 (0.24, 3.88) | 0.027* |
| MALE | 0 |  | 0 |  |
| AGE |  |  |  |  |
| > 35 YR | -4.39 (-18.19, 9.40) | 0.531 |  |  |
| 30 – 35 YR | 0.01 (-3.66, 3.68) | 0.997 |  |  |
| 25 – 30 YR | 0 |  |  |  |
| STATUS |  |  |  |  |
| MARRIAGE | 0.13 (-3.94, 4.20) | 0.95 |  |  |
| SINGLE | 0 |  |  |  |
| FAMILY BURDEN AS RESIDENT PERCEPTION |  |  |  |  |
| HIGH LEVEL | -3.99 (-8.25, 0.26) | 0.066 |  |  |
| MODERATE LEVEL | -1.29 (-3.26, 0.67) | 0.196 |  |  |
| LOW LEVEL | 0 |  |  |  |
| ALCOHOL |  |  |  |  |
| DRINKING | -0.33 (-2.48, 1.82) | 0.762 |  |  |
| NONE-DRINKING | 0 |  |  |  |
| SLEEP TIME |  |  |  |  |
| ≥ 35 H/WEEK | 2.85 (0.57, 5.13) | 0.014 | 2.41 (0.14, 4.67) | 0.038* |
| < 35 H/WEEK | 0 |  | 0 |  |
| EXERCISE TIME |  |  |  |  |
| > 5 H/WEEK | 3.12 (-1.62, 7.86) | 0.196 | 3.23 (-1.43, 7.90) | 0.173* |
| 1 − 5 H/WEEK | 2.22 (0.42, 4.03) | 0.016 | 2.25 (0.45, 4.05) | 0.015* |
| NEVER | 0 |  | 0 |  |

*p<0.05

Table 2. A univariate and multivariate analysis of the demographic factors and competence

| COMPETENCE | Univariate | | Multivariate | |
| --- | --- | --- | --- | --- |
|  | Coef. (95% CI) | P-value | Coef. (95% CI) | P-value |
| GENDER |  |  |  |  |
| FEMALE | 0.97 (-0.93, 2.87) | 0.316 |  |  |
| MALE | 0 |  |  |  |
| AGE |  |  |  |  |
| > 35 YR | -4.62 (-18.82, 9.57) | 0.522 |  |  |
| 30 – 35 YR | 1.11 (-2.67, 4.88) | 0.563 |  |  |
| 25 – 30 YR | 0 |  |  |  |
| STATUS |  |  |  |  |
| MARRIAGE | -0.09 (-4.29, 4.10) | 0.965 |  |  |
| SINGLE | 0 |  |  |  |
| FAMILY BURDEN AS RESIDENT PERCEPTION |  |  |  |  |
| HIGH LEVEL | -4.27 (-8.66, 0.13) | 0.057 |  |  |
| MODERATE LEVEL | -1.41 (-3.44, 0.62) | 0.172 |  |  |
| LOW LEVEL | 0 |  |  |  |
| ALCOHOL |  |  |  |  |
| DRINKING | 0.12 (-2.09, 2.33) | 0.914 |  |  |
| NONE-DRINKING | 0 |  |  |  |
| SLEEP TIME |  |  |  |  |
| ≥ 35 H/WEEK | 1.75 (-0.62, 411) | 0.147 |  |  |
| < 35 H/WEEK | 0 |  |  |  |
| EXERCISE TIME |  |  |  |  |
| > 5 H/WEEK | 1.67 (-3.22, 6.56) | 0.503 |  |  |
| 1 − 5 H/WEEK | 2.24 (0.38, 4.10) | 0.018 |  |  |
| NEVER | 0 |  |  |  |

*p<0.05

Table 3. A univariate and multivariate analysis of the demographic factors and relatedness

| RELATEDNESS | Univariate | | Multivariate | |
| --- | --- | --- | --- | --- |
|  | Coef. (95% CI) | P-value | Coef. (95% CI) | P-value |
| GENDER |  |  |  |  |
| FEMALE | 2.61 (0.48, 4.74) | 0.016 | 2.61 (0.49, 4.74) | 0.016* |
| MALE | 0 |  | 0 |  |
| AGE |  |  |  |  |
| > 35 YR | -1.90 (-17.96, 14.16) | 0.816 |  |  |
| 30 – 35 YR | 1.23 (-3.04, 5.51) | 0.57 |  |  |
| 25 – 30 YR | 0 |  |  |  |
| STATUS |  |  |  |  |
| MARRIAGE | 2.14 (-2.59, 6.87) | 0.374 |  |  |
| SINGLE | 0 |  |  |  |
| FAMILY BURDEN AS  RESIDENT PERCEPTION |  |  |  |  |
| HIGH LEVEL | -5.75 (-10.68, -0.82) | 0.023 | -5.48 (-10.37, -0.59) | 0.028* |
| MODERATE LEVEL | -2.29 (-4.56, -0.01) | 0.049 | -2.41 (-4.67, -0.16) | 0.036* |
| LOW LEVEL | 0 |  | 0 |  |
| ALCOHOL |  |  |  |  |
| DRINKING | -0.37 (-2.87, 2.13) | 0.769 |  |  |
| NONE-DRINKING | 0 |  |  |  |
| SLEEP TIME |  |  |  |  |
| ≥ 35 H/WEEK | 2.73 (0.06, 5.39) | 0.045 |  |  |
| < 35 H/WEEK | 0 |  |  |  |
| EXERCISE TIME |  |  |  |  |
| > 5 H/WEEK | 3.54 (-1.97, 9.05) | 0.207 |  |  |
| 1 − 5 H/WEEK | 2.80 (0.7, 4.89) | 0.009 |  |  |
| NEVER | 0 |  |  |  |

*p<0.05

Table 4. A univariate and multivariate analysis of the demographic factors and Grit

| GRIT | Univariate | | Multivariate | |
| --- | --- | --- | --- | --- |
|  | Coef. (95% CI) | P-value | Coef. (95% CI) | P-value |
| GENDER |  |  |  |  |
| FEMALE | 0.10 (-0.11, 0.31) | 0.345 |  |  |
| MALE | 0 |  |  |  |
| AGE |  |  |  |  |
| > 35 YR | 0.21 (-1.33, 1.76) | 0.785 |  |  |
| 30 – 35 YR | 0.22 (-0.19, 0.63) | 0.288 |  |  |
| 25 – 30 YR | 0 |  |  |  |
| STATUS |  |  |  |  |
| MARRIAGE | -0.06 (-0.52, 0.39) | 0.783 |  |  |
| SINGLE | 0 |  |  |  |
| FAMILY BURDEN AS  RESIDENT PERCEPTION |  |  |  |  |
| HIGH LEVEL | -0.09 (-0.57, 0.40) | 0.724 |  |  |
| MODERATE LEVEL | -0.11 (-0.33, 0.11) | 0.331 |  |  |
| LOW LEVEL | 0 |  |  |  |
| ALCOHOL |  |  |  |  |
| DRINKING | -0.03 (-0.27, 0.21) | 0.803 |  |  |
| NONE-DRINKING | 0 |  |  |  |
| SLEEP TIME |  |  |  |  |
| ≥ 35 H/WEEK | 0.11 (-0.14, 0.37) | 0.401 |  |  |
| < 35 H/WEEK | 0 |  |  |  |
| EXERCISE TIME |  |  |  |  |
| > 5 H/WEEK | 0.33 (-0.21, 0.86) | 0.231 |  |  |
| 1 − 5 H/WEEK | 0.18 (-0.03, 0.38) | 0.088 |  |  |
| NEVER | 0 |  |  |  |

*p<0.05

Table 5. A univariate and multivariate analysis of the demographic factors and Well-being

| WELL-BEING | Univariate | | Multivariate | |
| --- | --- | --- | --- | --- |
|  | Coef. (95% CI) | P-value | Coef. (95% CI) | P-value |
| GENDER |  |  |  |  |
| FEMALE | 0.75 (-0.28, 1.77) | 0.152 |  |  |
| MALE | 0 |  |  |  |
| AGE |  |  |  |  |
| > 35 YR | -5.27 (-12.93, 2.38) | 0.176 |  |  |
| 30 – 35 YR | 0.26 (-1.77, 2.30) | 0.8 |  |  |
| 25 – 30 YR | 0 |  |  |  |
| STATUS |  |  |  |  |
| MARRIAGE | -1.33 (-3.59, 0.93) | 0.247 |  |  |
| SINGLE | 0 |  |  |  |
| FAMILY BURDEN AS  RESIDENT PERCEPTION |  |  |  |  |
| HIGH LEVEL | -0.94 (-3.32, 1.43) | 0.435 |  |  |
| MODERATE LEVEL | -0.81 (-1.90, 0.29) | 0.148 |  |  |
| LOW LEVEL | 0 |  |  |  |
| ALCOHOL |  |  |  |  |
| DRINKING | -1.09 (-2.28, 0.09) | 0.071 |  |  |
| NONE-DRINKING | 0 |  |  |  |
| SLEEP TIME |  |  |  |  |
| ≥ 35 H/WEEK | 2.07 (0.81, 3.33) | 0.001 | 1.99 (0.77, 3.21) | 0.002 |
| < 35 H/WEEK | 0 |  | 0 |  |
| EXERCISE TIME |  |  |  |  |
| > 5 H/WEEK | 5.69 (3.13, 8.26) | <0.001 | 5.68 (3.16, 8.20) | <0.001 |
| 1 − 5 H/WEEK | 1.12 (0.14, 2.09) | 0.025 | 0.93 (-0.03, 1.90) | 0.058 |
| NEVER | 0 |  | 0 |  |

*p<0.05
